# Retraction: miR-96-5p enhances cell proliferation and invasion via targeted regulation of ZDHHC5 in gastic cancer

**DOI:** 10.1042/BSR-20191845_RET

**Published:** 2020-06-25

**Keywords:** gastric adenocarcinoma, prognosis, microRNA-96-5p, cell apoptosis

This article (*Bioscience Reports* (2020) 40(4), DOI: 10.1042/BSR20191845) is being retracted from *Bioscience Reports* at the request of the Editor-in-Chief and the Editorial Board following receipt of a notification from a reader, alerting the Editorial Board that the article had already been published in *World Journal of Gastroenterology* (DOI: 10.3748/wjg.v25.i47.6823), with a different authorship to the current paper.

The authors listed on the *Bioscience Reports* paper have been contacted in regard to the retraction, but have failed to respond.

